# Etiology and Clinical Characterization of Respiratory Virus Infections in Adult Patients Attending an Emergency Department in Beijing

**DOI:** 10.1371/journal.pone.0032174

**Published:** 2012-02-28

**Authors:** Xiaoyan Yu, Roujian Lu, Zhong Wang, Na Zhu, Wen Wang, Druce Julian, Birch Chris, Jianxin Lv, Wenjie Tan

**Affiliations:** 1 Key Laboratory for Laboratory Medicine of Ministry of Education, Institute of Medical Virology, Wenzhou Medical College, Wenzhou, Zhejiang, China; 2 National Institute for Viral Disease Control and Prevention, China CDC, Beijing, China; 3 Peking Union Medical College Hospital, CAMS, Beijing, China; 4 Virology Department, Victorian Infectious Diseases Reference Laboratory, North Melbourne, Victoria, Australia; Erasmus Medical Center, The Netherlands

## Abstract

**Background:**

Acute respiratory tract infections (ARTIs) represent a serious global health burden. To date, few reports have addressed the prevalence of respiratory viruses (RVs) in adults with ARTIs attending an emergency department (ED). Therefore, the potential impact of respiratory virus infections on such patients remains unknown.

**Methodology/Principal Findings:**

To determine the epidemiological and clinical profiles of common and recently discovered respiratory viruses in adults with ARTIs attending an ED in Beijing, a 1-year consecutive study was conducted from May, 2010, to April, 2011. Nose and throat swab samples from 416 ARTI patients were checked for 13 respiratory viruses using multiple reverse transcription polymerase chain reaction(RT-PCR) assays for common respiratory viruses, including influenza viruses (Flu) A, B, and adenoviruses (ADVs), picornaviruses (PICs), respiratory syncytial virus (RSV), parainfluenza viruses (PIVs) 1–3, combined with real-time RT-PCR for human metapneumovirus (HMPV) and human coronaviruses (HCoVs, -OC43, -229E, -NL63, and -HKU1). Viral pathogens were detected in 52.88% (220/416) of patient samples, and 7.21% (30/416) of patients tested positive for more than one virus. PICs (17.79%) were the dominant agents detected, followed by FluA (16.11%), HCoVs (11.78%), and ADV (11.30%). HMPV, PIVs, and FluB were also detected (<3%), but not RSV. The total prevalence and the dominant virus infections detected differed significantly between ours and a previous report. Co-infection rates were high for HCoV-229E (12/39, 30.76%), PIC (22/74, 29.73%), ADV (12/47, 25.53%) and FluA (15/67, 22.39%). Different patterns of clinical symptoms were associated with different respiratory viruses.

**Conclusions:**

The pattern of RV involvement in adults with ARTIs attending an ED in China differs from that previously reported. The high prevalence of viruses (PIC, FluA, HCoVs and ADV) reported here strongly highlight the need for the development of safe and effective therapeutic approaches for these viruses.

## Introduction

Viruses cause most respiratory tract infections, yet the specific infectious agent often remains unknown [1], [2]. Comparison of the viral causes of infection provides a useful starting point for an understanding of illness following respiratory infection. It also provides data relevant to the development of prevention strategies. The following viruses (in no particular order) have been detected during acute respiratory infections (ARIs) [1], [2]: influenza virus (Flu), parainfluenza virus (PIV), adenovirus (ADV), picornavirus (PIC, including rhinovirus and enterovirus), respiratory syncytial virus (RSV), human metapneumovirus (hMPV), and human coronavirus (HCoV). Respiratory virus infections are diagnosed in four principal ways: virus culture, serology, immunofluorescence/antigen detection, and nucleic acid/PCR-based tests. Nucleic acid tests are significantly more sensitive than the other methods described, which may have an impact on the viruses detected [1], [2]. Nucleic acid tests are now being multiplexed, allowing rapid simultaneous detection of many viruses [1], [2].

In China, several groups have reported the prevalence and clinical presentation of viral infections [3]–[5], particularly those of HCoV infections by reverse transcriptase PCR (RT-PCR) assays performed on clinical specimens taken from adults with ARTIs from 2005 to 2009 in Beijing [5]. However, more precise data regarding their epidemiology and clinical characteristics are lacking in mainland China after the 2009 H1N1 pandemic. Moreover, to the best of our knowledge, there is no published report that describes the potential impact of viral agents on adults with ARTIs admitted to an ED in China. To directly address this situation, we screened for the presence of 13 respiratory virus in adults with ARTI admitted to Peking Union Medical College Hospital from May, 2010, to April, 2011, in an effort to gain a better understanding of the seasonality, epidemiology, and clinical profile of these viruses in a city with a population of more than 22 million.

## Materials and Methods

### Ethics Issues

All aspects of the study were performed in accordance with the national ethics regulations and approved by the Institutional Review Boards of the Centre for Disease Control and Prevention of China, as well as the Ethics Committee of Peking Union Medical College Hospital. Participants were recieved “Written Informed Consent” on the study's purpose and of their right to keep information confidential. Written consent was obtained from all participants or their guardians.

### Patients and Specimens

From May, 2010, to April, 2011, 416 nasal and throat swabs (NTS) were collected from patients with acute respiratory symptoms who had been admitted to the ED at Peking Union Medical College Hospital, Beijing, China. Patients were provided written informed consent before specimen collection and testing. Patients over 14 years of age were selected according to a set of criteria that included respiratory symptoms, a body temperature above 37.5°C, and a normal or low leukocyte count, but not pulmonary abnormalities on radiography [4]. Symptoms, history of illness, results of a clinical examination and laboratory investigations, and demographic data were recorded for each patient, using a standardized form. Clinical information of patients with virus infection was reviewed retrospectively from the records. Swabs were kept in viral transport medium and stored at −70°C prior to analysis.

### Nucleic Acid Extraction and cDNA Synthesis

Nucleic acid was extracted using QIAamp MiniElute Virus Spin kits (Qiagen, Mississauga, Ontario, Canada) according to the manufacturer's instructions. cDNA was synthesized from 10 µL RNA eluate using random hexamer primers and AMV Reverse Transcriptase (Promega, Madison, WI), as described previously [6]–[8].

### Detection of Common Respiratory Viruses by RT-PCR

All samples were tested by multiple nested RT-PCR screening for common respiratory viruses infections (Table 1), including influenza virus types A, B, adenovirus (group 1), PIC (enterovirus, rhinovirus), respiratory syncytial virus (group 2), and parainfluenza virus types 1–3 (group 3), as described previously [6]. The analytic sensitivity of PCR or RT-PCR for detection of single virus is 10–100 molecules. All PCR products were confirmed by sequencing.

**Table 1:** Details of assays used to identify RVs.

Assays and		Sequences* of primers** and probes***		Target genes	Thermal profiles	Ref
viruses detected						
**Multiple-nested PCR**						
**Mix1**	Flu A	FA-1F	CAGAGACTTGARRATGTYTTTGC	Matrix	**1st round:** 48°C for 45 min; 94°C for 2 min;94°C for 30 s, 55°C for 30 s, 72°C for 60 s,35 cycles; 72°C for 5 min.**2nd round:** 94°C for 5 min; 94°C for 30 s, 55°C for 30 s, 72°C for 60 s, 25 cycles; 72°C for 5 min.	[6]
		FA-1R	GGCAAGYGCACCRGYWGARTARCT			
		FA-2F	GACCRATCCTGTCACCTCTGACT			
		FA-2R	AYYTCYTT GC CCATGGAATGT			
	Flu B	FB-1F	GTGACTGGTGTGATACCACT	HA		
		FB-1R	TGTTTTCACCCATATTGGGC			
		FB-2F	CATTTTGCAAATCTCAAAGG			
		FB-2R	TGGAGGCAATCTGCTTCACC			
	ADV	AD-1F	GCCGCAGTGGTCTTACATGCACATC	Hexon		
		AD-1R	CAGCACGCCGCGGATGTCAAAGT			
		AD-2F	GCCACCGAGACGTACTTCAGCCTG			
		AD-2R	TTGTACGAGTACGCGGTATCCTCGCGGTC			
		AD-2F′	CMGASACSTACTTCAGYMTG			
		AD-2R′	GTASGYRKTRTCYTCSCGGTC			
**Mix2**	RSV	RS-1F	TGGGAGARGTRGCTCCAGAATACAGGC	N	**1st round:** same as Mix1;**2nd round:** 94°C for 5 min; 94°C for 30 s, 50°C for 30 s, 72°C for 60 s, 25 cycles; 72°C for 5 min.	[6]
		RS-1R	ARCATYACTTGCCCTGMACCATAGGC			
		RS-2F	ACYAAATTAGCAGCAGGG			
		RS-2R	CTCTKGTWGAWGATTGTGC			
	Piconavirus	PIC-1F	GCACTTCTGTTTCCCC	5′-UTR		
		PIC-1R	CGGACACCCAAAGTAG			
		PIC-2F	GCACTTCTGTTTCCCC			
		PIC-2R	GCATTCAGGGGCCGGAG			
**Mix3**	PIV (-1,-2,-3)	P123-1F	GTWCAAGGAGAYAATCARGC	L	**Same as Mix1 profile**	[6]
		P123-1R	GRTCYGGAGTTTCWARWCC			
		P1-2F	GCATCAGACCCTTATTCATG			
		P1-2R	GTTGTATCAAGCATCCCGGC			
		P2-2F	CAGCCGATCCATACTCATTG			
		P2-2R	CTTGTGGTGTCAAAAAATCC			
		P3-2F	GCTGTTACTACAAGAGTACC			
		P3-2R	GTTGCCAGATTTGAGGATGC			
**Real- Time rtPCR**						
	HMPV	HMP-F	CATATAAGCATGCTATATTAAAAGAGTCTC	N	48°C 30 min;95°C for 15 min;95°C for 15 s,68°C for 1 min,45cycles.	[8]
		HMP-R	CCTATTTCTGCAGCATATTTGTAATCAG			
		HMP-P	FAM-TGYAATGATGAGGGTGTCACTGCGGTTG-TAMRA			
	HCoV-OC43	OC43-F	GCTCAGGAAGGTCTGCTCC	N		[7]
		OC43-R	TCCTGCACTAGAGGCTCTGC			
		OC43-P	FAM -TTCCAGATCTACTTCGCGCACATCC- TAMRA			
	HCoV-229E	229E- F	CGCAAGAATTCAGAACCAGAG	N		[7]
		229E-R	GGCAGTCAGGTTCTTCAACAA			
		229E-P	FAM -CCACACTTCAATCAAAAGCTCCCAAATG- TAMRA			
	HCoV-NL63	NL63-F	AGGACCTTAAATTCAGACAACGTTCT	N		[7]
		NL63-R	GATTACGTTTGCGATTACCAAGACT			
		NL63-P	FAM-TAACAGTTTTAGCACCTTCCTTAGCAACCCAAACA- TAMRA			
	HCoV-HKU1	HKU-F	AGTTCCCATTGCTTTCGGAGTA	N		[7]
		HKU-R	CCGGCTGTGTCTATACCAATATCC			
		HKU-P	FAM -CCCCTTCTGAAGCAA- MGB			

*K = G+T,M = A+C,R = A+G,S = G+C,W = A+T,Y = C+T.

**1st round primers:-1F,-1R; 2 nd round primers: -2F or -2F′, -2R or -2R′.

***Labeled at 5′ end with FAM and terminally quenched at the 3′ end with TAMRA or MGB.

### Detection of HMPV and HCoV by Real-Time RT-PCR

Specimens were tested for human metapneumovirus (HMPV) and non-SARS HCoVs (including OC43, 229E, NL63, and HKU1) by real-time RT-PCR(Table 1) using a TaqMan RNA-to-CT 1-Step kit (Applied Biosystems, USA) and an ABI Prism 7000 TaqMan machine (Applied Biosystems, USA), as described previously [7], [8]. The lower limit of detection of each real-time RT PCR assay was 100 copies/20 µL, with intra-assay coefficients of variability (CVs) between 0.45% and 1.02% (*n* = 3), and inter-assay CVs of 0.68–2.24%.

### Statistical Analysis

Age, maximum body temperature, laboratory parameters, clinical features, and annual incidence of each virus were compared using the χ^2^-test or Fisher's exact test for categorical variables, and the data sets were compared between groups using a two-tailed paired Student's t-test to obtain P values. *P*<0.05 was considered significant.

## Results

### Viral Prevalence

Of 416 nasopharyngeal samples analysed by both multiple nested RT-PCR and real-time RT-PCR (Table 1), 220 (52.88%) were positive for one or more agents, comprising 47.51% (86/181) males and 57.02% (134/235) females, (χ^2^ = 3.709; *P*>0.05). Detection of any viral agent was significantly more common during the summer (July and August, 2010) and winter (December 2010 and January 2011) in patients aged 20–50 years old. As shown in Table 2, PIC had the highest detection rate (17.79%, 74/416), followed by FluA (16.11%, 67/416), HCoVs (HCoV-OC43, 0.96%; HCoV-229E, 9.38%; HCoV-NL63, 0.48%; HCoV-HKU1, 0.96%; total 11.78%, 49/416), ADV (11.30%, 47/416), hMPV (2.16%, 9/416), PIV (0.96%, 4/416), and FluB (0.72%, 3/416). No RSV was detected, and 196 samples (47.11%) were negative for all 13 viruses.

**Table 2:** Respiratory viruses detected in 416 adults patients attending an emergency department.

Virus	No. of samples											Rate (%)
	FluA	FluB	ADV	RSV	Picornavirus	PIVs	HMPV	HCoV-OC43	HCoV-229E	HCoV-NL63	HCoV-HKU1	
FluA	**67**	0	2	0	8	0	2	0	5	0	0	16.11
FluB		**3**	1	0	1	0	0	0	0	0	0	0.72
ADV			**47**	0	5	0	0	1	3	0	0	11.3
RSV				**0**	0	0	0	0	0	0	0	0
Picornavirus					**74**	1	0	0	6	0	1	17.79
PIVs						**4**	0	0	1	0	0	0.96
HMPV							**9**	0	0	0	0	2.16
HCoV-OC43								**4**	0	0	0	0.96
HCoV-229E									**39**	0	0	9.38
HCoV-NL63										**2**	0	0.48
HCoV-HKU1											**4**	0.96
1 virus	52	1	35	0	52	2	7	3	27	2	3	
2 viruses	12	2	10	0	20	2	2	1	10	0	1	
3 viruses	3	0	2	0	2	0	0	0	2	0	0	

### Seasonal distribution

**Figure 1:**
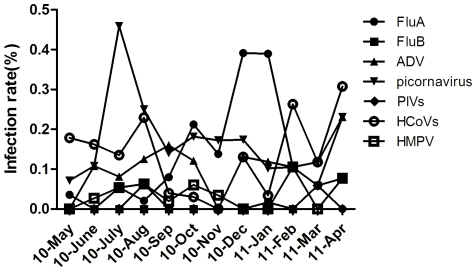
Seasonal variation in individual virus infection.

The seasonal distribution of detection varied during the period from May, 2010, to April, 2011 (Figures 1). FluA and PIC exhibited remarkable seasonal distributions. Peaks in FluA detection occurred in winter (Figure 1), with a detection rate of 41.03% (19/47) in December, 2010, and 38.98% (23/59) in January, 2011. Furthermore, peak PIC activity occurred in July and August of 2010, with detection rates of 43.24% (16/37) and 22.92% (11/48). The lowest (≤10%) detection occurred in May and September, 2010, and March, 2011.

### Co-infection of respiratory viruses

Of the 416 patients, 30 were co-infected with at least two respiratory viruses, with detection rates of 7.21% (30/416) of total samples and 14.63% (30/220) of positive samples. Most co-infected patients were 20–39 years of age (66.67%, 20/30), and PIC was frequently detected co-infecting virus (29.73%, 22/74) (Table 2), most commonly with FluA (27.5%, 8/22). In addition, the co-detection rates of FluA, ADV, and HCoV-229E were common as 22.39% (15/67), 25.53% (12/47) and 30.76% (12/39), respectively.

### Clinical profiles associated with FluA, ADV, PIC, and HCoV infection

The clinical characteristics of patients with FluA, ADV, PIC, and HCoV infection are summarized in Table 3. All patients infected with these viruses presented with respiratory infection symptoms including fever, cough, headache, sore throat, runny nose, and so on; a few also presented with diarrhoea. The three most common symptoms of infection with FluA were cough (82.09%), sore throat (79.10%), and headache (65.67%). The most common symptoms of infection with ADV, PIC, and HCoV were headache, sore throat, and cough, respectively. Most infected patients were aged 20–49 years old, and slightly more were female.

**Table 3:** Comparisons of characteristics of patients with FluA, Adv, PIC, and HCoV infection.

Parameter	No. of events (*n* = 416 adults; %)				
	FluA	ADV	Picornavirus	HCoVs	*P value*
	(*n* = 67)	(*n* = 47)	(*n* = 74)	(*n* = 49)	
**Age (total)**					
≤19 y (33)	3(4.48)	6(12.77)	10(13.51)	4(8.16)	
20–49 y (305)	48(71.64)	33(70.21)	55(74.32)	39(79.59)	
≥50 y (76)	16(23.88)	8(17.02)	8(10.81)	6(12.24)	
**Gender(M/F)**	26/41	20/27	35/39	14/35	
**Fever >39°C**	22(32.84)	9(19.15)	21(28.38)	19(38.78)	0.188
**Clinical symptoms**					
Headache	44(65.67)	38(80.85)	63(85.14)	38(77.55)	0.007
Sore throat	53(79.10)	25(53.19)	54(72.97)	29(59.18)	0.02
Cough	55(82.09)	17(36.17)	48(64.86)	15(30.61)	<0.001
Rhinorrhoea	41(61.19)	14(29.79)	37(50.00)	16(32.65)	0.002
Expectoration	37(55.22)	13(27.66)	36(48.65)	12(24.49)	0.004
Rigors	22(32.84)	11(23.40)	28(37.84)	17(34.69)	0.422
Nasal obstruction	26(38.81)	8(17.02)	30(40.54)	9(18.37)	0.007
Diarrhoea	1(1.49)	2(4.26)	1(1.35)	3(6.12)	0.316

## Discussion

RVs, a major cause of ARTIs [1]–[9], which cause significant global human morbidity and mortality, especially in infants, are a serious health and economic burden and have become a national public health detection and monitoring priority. This study focused on the distribution of human respiratory viruses in adult patients with ARTI who had been admitted to the ED at Peking Union Medical College Hospital, Beijing, China, at the end of the H1N1 pandemic (May 2010 to April 2011). We determined the prevalence of 13 respiratory viruses, and analysed the clinical profiles of the four most common (PIC, FluA, ADV, HCoVs). This study provides useful information on the prevalence, clinical profiles, and epidemiology of specific viral aetiology in adults with ARTI attending an ED in China, and will contribute to the diagnosis, treatment, and prevention of ARTI in adults.

We collected nasal and throat swab samples from adult patients attending an ED in Beijing, and analysed them using both multiple nested RT-PCR and real-time RT-PCR methods to detect 13 human RVs: FluA, B, RSV, PIVs 1–3, PIC, ADV, HCoVs (-229E, -OC43, -NL63, -HKU1), and hMPV. Of the 416 samples, 220 (52.88%) contained at least one virus, which is consistent with other studies performed worldwide (34.6–62.6%) [1]–[14], but a little higher than a previous report from Beijing [4]. This may be due to differences in detection methods, population, and/or the time the study was performed. This also reminds us that in the year after influenza pandemics, the frequency distribution of these 13 respiratory viruses in adult patients in the Beijing area may increase, pending further studies. In addition, of the 416 samples, no viruses were detected in 196 (47.11%). This suggests the existence of other pathogens, such as human parvovirus [15] and human polyomavirus [16], [17], or unknown others. Thus, further optimization of specimen collection (location, period) and detection methods may improve the detection rate.

By comparing the patterns of detection of the four most frequently detected viruses (PIC, FluA, ADV, HCoVs), gender, and age group, we found similar detection rates in males and females, and those aged 20–49 had highest prevalence (70%), which is consistent with trends of RV infection reported previously [1]–[9].

PICs, including rhinovirus and enterovirus, are frequently detected in patients with upper respiratory tract infections [18], [19]. A previous study showed that 50% of common cold infections are caused by rhinovirus [18]. PIC had the highest detection rate (17.79%, 74/416), and covered all age groups. Peaks occurred in August and September, 2010, as in previous reports [18]. This PIC detection rate is higher than that reported elsewhere [4], likely due to the fact that the previous study focused only on rhinovirus and enterovirus, neglecting the other PICs that may cause respiratory infection. In addition, different sample collection periods and detection methods may have contributed to the discrepancies in detection rates [10].

The detection rate of FluA, the most common respiratory virus, was 16.11% with significant seasonality. Peaks appeared in winter, and no FluA was detected in June, 2010, and April, 2011. Because this study was conducted at the end of the H1N1 pandemic, many people had recently been vaccinated and so possessed high levels of circulating antibody, likely causing a decline in infection rate. Other reports have indicated that FluA infection is interfered with by rhinovirus infection [19], [20], which may also have decreased the detection rate. Typing and identification of seasonal influenza virus and H1N1 influenza virus warrant further research.

Epidemiological studies have shown that more than 15% of common colds in adults are caused by human coronavirus (HCoV) [18], with HCoV- 229E and -OC43 being the most common [18]. The detection rate was 11.78% in this study, significantly higher than in previous reports [5]. All four non-SARS HCoVs (-OC43, -229E, -NL63, and -HKU1) were detected, the highest being HCoV-229E (9.38%), which differs from the data of Ren and co-workers [5]. This was likely due to different detection methods or the peak in HCoV-OC43 infection every 2–3 years [21]. The two HCoVs identified most recently, HCoV-NL63 and HCoV-HKU1 [22], [23], had low detection rates (<1%), similar to HCoV-OC43, but markedly lower than that of HCoV-229E.

ADV, an important pathogen in infants, was also detected frequently (11.3%) in this study. HMPV, which usually infects children [24], [25], was detected in 2.16% of adult patients in this study. The etiological significance of HMPV infection in adult ARTI patients requires further study. PIVs were detected only infrequently (0.96%), markedly lower than in previous reports [4]. Several factors may account for this disparity: we detected mainly PIV1–3, but PIV4 was in reality the most common virus; or the PIV-4 infection rate increased in recent outbreaks of ARTIs [26]. In addition, PIV causes mainly LRTIs, but samples in this study were mainly collected from patients with URTIs. RSV, another important viral agent in infants and young children, was not detected in this study.

Of the 220 positive patients with RV infection, 30 were infected with more than one viral agent, 27 of which represented co-infections and 3 of which were triple-infections. Twenty-two PIC-positive patients were co-infected with FluA, ADV, 229E, PIV, HKU1, or FluB. Rhinovirus is a major etiological agent of URTIs, and is implicated in 25–70% of co-infections in hospitalized patients [18]. Therefore, further identification and typing of PIC is needed to elucidate the pathogenesis and clinical significance of rhinovirus and enterovirus.

Consistent with previous reports [1], [2], [4]–[7], [18], patients infected with the four most frequently detected viruses (FluA, ADV, PIC, HCoVs) presented with obvious symptoms of respiratory infection, including headache, sore throat, cough, runny nose, chills, and coryza. Except for chills and diarrhoea (*P*>0.05), the clinical characteristics of ARTIs caused by these four viruses differed slightly. Headache was associated with PIC, ADV, and HCoV infection. Cough was significantly more common in influenza patients; and sore throat, cough, rhinorrhoea, nasal symptoms were more common in those infected with PIC, which were in line with previous reports [2], [6], [18]. Molecular assays are useful for diagnosis of RTIs, because it is often difficult even for the experienced clinician to pinpoint the aetiological agent based only on clinical information. Thus, etiological research may facilitate physicians' appropriate antibiotic use, and will provide useful information for surveillance and control of ARTIs [27].

In summary, we conducted a comprehensive analysis of the potential impact of 13 RVs in adults with ARTIs admitted to an ED in Beijing. Our study is original and unique in several aspects. First, to the best of our knowledge, it is the first use of sensitive, real-time RT-PCR assays targeting all four HCoVs and hMPV in adults with ARTIs in China. Compared to the reports of Ren et al. [4], [5] regarding adult ARTIs which specimens collected from the same hospital (Table 4), the detection rates of several aetiological agents (hMPV, total HCoVs, HCoV-229E, and HCoV-HKU1) in this study were significantly higher (P<0.05,the data were compared between groups of specimens for virus detected using a Student's *t*-test), although the reasons for this require further investigation. The second unique aspect of this study is the identification of four leading viral agents (PIC, FluA, ADV, and HCoVs) and associated clinical profiles in adults with ARTIs admitted to the ED. The pattern differed from those in previous reports from the same area. To the best of our knowledge, this is the first report of the potential impact of four viruses (PIC, FluA, ADV, and HCoVs) as causes of ARTIs in adults admitted to an ED in China. The main limitation of the study is that we did not evaluate other recently identified viruses (such as bocavirus, WU, and KI virus) or atypical aetiologies. An additional limitation is that the subjects were recruited only in one continuous year. It is possible that these limitations might have affected the results and should be taken into consideration.

**Table 4:** Comparison of detection rates of three studies of adults with ARTI in Beijing.

Virus	Positive detection rate			
	Ren L^4^	Ren L^5^	Yu X	*P value*
	(2005–2007)	(2005–2009)	(2010–2011)	
Total	2010/5808(34.6%)		220/416(52.88%)	<0.001
Flu(A+B+C/(A+B)	1119/5808(19.3%)		67/416(16.11%)	0.113
ADV	51/5808(0.9%)		47/416(11.30)	<0.001
PIVs	252/5808(4.3%)		4/416(0.96%)	0.001
Picornavirus/HRVs+EVs	564/5808(9.7%)		74/416(17.79%)	<0.001
RSV	30/5808(0.5%)		0/416(0%)	0.27
HMPV	19/5808(0.3%)		9/416(2.16%)	<0.001
HCoVs	65/5808(1.1%)		49/416(11.79%)	<0.001
HCoV-OC43		50/8396(0.6%)	4/416(0.96%)	0.541
HCoV-229E		15/8396(0.2%)	39/416(9.38%)	<0.001
HCoV-NL63		14/8396(0.2%)	2/416(0.48%)	0.173
HCoV-HKU1		8/8396(0.1%)	4/416(0.96%)	0.002

## References

[1] Mahony JB (2010). Nucleic acid amplification-based diagnosis of respiratory virus infections.. Expert Rev Anti Infect Ther.

[2] Beck ET, Henrickson KJ (2010). Molecular diagnosis of respiratory viruses.. Future Microbiol.

[3] 1He J, Gong Y, Zhong WJ, Xu L, Liu Y (2011). Study on the viral etiology of acute respiratory tract infections in the Shanghai area during 2009–2010.. J Microbes Infect.

[4] Ren L, Gonzalez R, Wang Z, Xiang Z, Wang Y (2009). Prevalence of human respiratory viruses in adults wi[]th acute respiratory tract infections in Beijing, 2005–2007.. Clin Microbiol Infect.

[5] Ren L, Gonzalez R, Xu J, Xiao Y, Li Y (2011). Prevalence of human coronaviruses in adults with acute respiratory tract infections in Beijing, China.. J Med Virol.

[6] Druce J, Tran T, Kelly H, Kays M, Chibo D (2005). Laboratory diagnosis and surveillance of human respiratory viruses by PCR in Victoria, Australia, 2002–2003.. J Med Virol.

[7] Esposito S, Bosis S, Niesters HG, Tremolati E, Begliatti E (2006). Impact of human coronavirus infections in otherwise healthy children who attended an emergency department.. J Med Virol.

[8] Maertzdorf J, Wang CK, Brown JB, Quinto J D, Chu M (2004). Real-time reverse transcriptase PCR assay for detection of human metapneumoviruses from all known genetic lineages.. J Clin Microbiol.

[9] Bharaj P, Sullender WM, Kabra SK, Mani K, Cherian J (2009). Respiratory viral infections detected by multiplex PCR among pediatric patients with lower respiratory tract infections seen at an urban hospital in Delhi from 2005 to 2007.. Virol J.

[10] Brittain-Long R, Westin J, Olofsson S, Lindh M, Andersson LM (2009). Prospective evaluation of a novel multiplex real-time PCR assay for detection of fifteen respiratory pathogens-duration of symptoms significantly affects detection rate.. J Clin Virol.

[11] Brittain-Long R, Andersson LM, Olofsson S, Lindh M, Westin J (2011). Seasonal variations of 15 respiratory agents illustrated by the application of a multiplex polymerase chain reaction assay.. Scan J Infect Dis.

[12] Lam WY, Yeung AC, Tang JW, Ip M, Chan EW, Hui M (2007). Rapid multiplex nested PCR for detection of respiratory viruses.. J Clin Microbiol.

[13] Berkley JA, Munywoki P, Ngama M, Kazungu S, Abwao J (2010). Viral etiology of severe pneumonia among Kenyan young infants and children.. JAMA.

[14] Kim C, Ahmed JA, Eidex RB, Nyoka R, Waiboci LW (2011). Comparison of nasopharyngeal and oropharyngeal swabs for the diagnosis of eight respiratory viruses by real-time reverse transcription-PCR assays.. PLoS One.

[15] Allander T, Tammi MT, Eriksson M, Bjerkner A, Tiveljung-Lindell A (2005). Cloning of a human parvovirus by molecular screening of respiratory tract samples.. Proc Natl Acad Sci.

[16] Allander T, Andreasson K, Gupta S, Bjerkner A, Bogdanovic G (2007). Identification of a third human polyomavirus.. J Virol.

[17] Gaynor AM, Nissen MD, Whiley DM, Mackay IM, Lambert SB (2007). Identification of a novel polyomavirus from patients with acute respiratory tract infections.. PLoS Pathogen.

[18] Greenberg SB (2011). Update on rhinovirus and coronavirus infections.. Semin Resoir Crit Care Med.

[19] Greer RM, McErlean P, Arden KE, Faux CE, Nitsche A (2009). Do rhinoviruses reduce the probability of viral co-detection during acute respiratory tract infections?. J Clin Virol.

[20] Linde A, Rotzen-Ostlund M, Zweygberg-Wirgart B, Rubinova S, Brytting M (2009). Does viral interference affect spread of influenza?. Euro Surveill.

[21] Gaunt ER, Hardie A, Claas ECJ, Simmonds P, Templeton KE (2010). Epidemiology and clinical presentation of the four human coronaviruses 229E, HKU1,1NL63, and OC43 detected over 3 years using a novel multiplex real-time PCR method.. J Clin Microbiol.

[22] van der Hoek L, Pyrc K, Jebbink MF, Vermeulen-Oost W, Berkhout RJ (2004). Identification of a new human coronavirus.. Nat Med.

[23] Woo PC, Lau SK, Chu CM, Chan KH, Tsoi HW (2005). Characterization and complete genome sequence of a novel coronavirus, coronavirus HKU1, from patients with pneumonia.. J Virol.

[24] Van den Hoogen BG, de Jong JC, Groen J, Kuiken T, de Groot R (2001). A newly discovered human pneumovirus isolated from young children with respiratory tract disease.. Nat Med.

[25] Schildgen V, van den Hoogen B, Fouchier R, Tripp RA, Alvarez R (2011). Human Metapneumovirus: Lessons Learned over the First Decade.. Clin Microbiol Rev.

[26] Ren L, Gonzalez R, Xie Z, Xiong Z, Liu C (2011). Human parainfluenza virus type 4 infection in Chinese children with lower respiratory tract infections: a comparison study.. J Clin Virol.

[27] Brittain-Long R, Westin J, Olofsson S, Lindh M, Andersson LM (2011). Access to a polymerase chain reaction assay method targeting 13 respiratory viruses can reduce antibiotics: a randomized, controlled trial.. BMC Med.

