# Correction: Etiology and Clinical Characterization of Respiratory Virus Infections in Adult Patients Attending an Emergency Department in Beijing

**DOI:** 10.1371/annotation/037611b5-b1fc-40a0-86f3-c9e65a9b90f5

**Published:** 2012-06-12

**Authors:** Xiaoyan Yu, Roujian Lu, Zhong Wang, Na Zhu, Wen Wang, Druce Julian, Birch Chris, Jianxin Lu, Wenjie Tan

There was an error in the eighth author's name. Jianxin Lu is correct. 

